# Study on creep characteristics and component model of saline soil in hexi corridor

**DOI:** 10.1038/s41598-023-42548-6

**Published:** 2023-10-23

**Authors:** Yun-yan Yu, Chong-liang Luo, Wen-hao Cui, Jing-yan Tao, Ting-hua Zhang

**Affiliations:** 1 School of Civil Engineering, Lanzhou Jiaotong University, Lanzhou, 730070 China; 2 School of Civil Engineering, Tianshui Normal University, Tianshui, 741000 China

**Keywords:** Civil engineering, Sedimentology

## Abstract

Accurate mastery of the creep characteristics of unsaturated saline soil is extremely important for the long-term stability and safe operation of all types of buildings. In this paper, the research object focused on the saline soil of the Zhangye area, Hexi corridor. The indoor triaxial CU creep test was carried out by means of graded loading to study the creep characteristics of saline soil under different salt content and loading stress. The Merchant and Burgers models were used to predict the creep behavior of the saline soils, and the predicted results were compared with the experimental values. The results showed that the triaxial creep curve of saline soil developed in stage III. Namely, transient creep stage, deceleration creep stage and steady-state creep stage. The creep deformation increases with the increase of salt content and loading stress. The stress–strain isochronous curve has non-linear growth, and the cluster of curves develops from dense to sparse after increasing to long-term strength (100∼150 kPa). The parameters of the Merchant and Burgers model vary with salt content and loading stress, and the creep curve predicted by the Burgers model is closer to the test value.

## Introduction

Saline soil is a general term for the saline-alkali soil, which is widely distributed in the inland and coastal areas of China. Among them, inland saline soil accounts for 69.03% of the total area of saline soil in China, and it is unsaturated in the complex arid climate environment of inland. With the advancement of the Belt and Road initiative and the transportation power strategy, lots of infrastructures have been built on inland saline land. However, saline soil belongs to a special soil, which has the characteristics of salt expansion, collapse, corrosion and creep. It will lead to frequent engineering diseases in saline soil area ^1,2^, which seriously affects the durability and safe operation of buildings and structures. It has been found that the subgrade and foundation will undergo triaxial creep deformation under the action of traffic load. The accumulation of deformation leads to uneven settlement of subgrade and foundation, which is easy to induce diseases such as track and pavement damage ^3,4^. Therefore, it is of great theoretical value and engineering significance to study the triaxial creep behavior and the creep model of saline soil.

Currently, scholars have conducted a lot of test research on soil creep characteristics and models. The creep test is an important means to reveal the creep deformation characteristics of the rock and soil. Yuan et al. ^5^ conducted triaxial consolidation creep tests on coastal soft soils and proposed a method to determine the parameters of the Merchant model associated with the confining pressure and stress level. Wang et al. ^6^ conducted a triaxial creep test on a slope loess in Shanxi Province, and analyzed the influence of loading time and deviatoric stress on the creep characteristics of loess. Liu et al. ^7^ analyzed the creep characteristics of saturated Q2 loess by the uniaxial creep test. The creep strength is 75∼80% of the unconfined compressive strength, and the creep behavior can be simulated by Burgers model Cai et al. ^8^ studied the effects of different densities, gradation types and loading methods on the creep characteristics of saturated sand through confined high-pressure uniaxial compression tests, and proposed a one-dimensional creep model and parameters that conform to sand. Wang et al. ^9^, Zhao et al. ^10^ studied the triaxial creep characteristics of loess under different confining pressures and different matric suction conditions, and found that both confining pressures and matric suction have significant effects on the creep characteristics, and the creep of loess experienced three processes: attenuating creep, stable creep, and accelerated creep. Xue et al. ^11^ carried out triaxial CD creep tests on the red clay in Chongqing under different confining pressures, compared three different existing theoretical models, and established a new empirical model based on the test results. Long et al. ^12^ conducted triaxial creep tests on compacted red clay using the SLB-1 triaxial shear permemeter, and studied the creep characteristics of red clay under different confining pressures, loading stresses, and drainage conditions. Meanwhile, a new creep model is also established; compared with Burgers model and Singh-Mitchell model, the calculated curve of the new creep model is in good agreement with the experimental curve. Wang et al. ^13^ revealed the influences of the low confining pressure on the creep characteristics of saturated the red mudstone by triaxial creep test, and proposed a new extended Burgers model of Newtonian body. Zhou and Wang ^14,15^ prepared saline soil of NaCl and Na_2_SO_4_ for Lanzhou loess after salt washing and studied the consolidation creep characteristics and the creep model of saturated saline soil with different salt contents. Previous investigations have mainly focused on the triaxial creep tests of special soils such as soft soil, loess, sand and red clay soil; however, fewer studies have been reported on the triaxial creep properties and models of special unsaturated saline soils.

With the continuous construction of engineering construction facilities in inland saline soil areas in China, engineering accidents have also increased rapidly ^16,17^. Accurately understanding the creep behavior of unsaturated saline soil is the key to curb engineering accidents and reduce engineering risks in saline soil areas. In view of this, this article investigates the creep properties and creep models of unsaturated saline soils under different salt contents based on triaxial CU creep tests, obtains triaxial creep curves and stress–strain isochronous curve relationships, and analyzes the factors and mechanisms that influence creep properties. At the same time, the Merchant and Burgers models are established in a three-dimensional state and the model parameters are identified to explore the applicable triaxial creep model for unsaturated saline soils.

## Triaxial test of saline soil

### Mechanical parameter test

**Figure 1:**
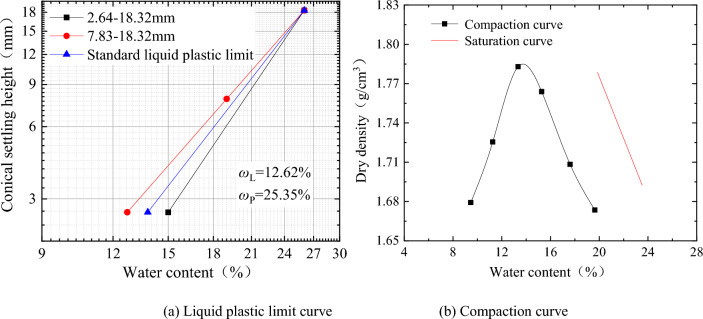
Test results of soil physical and mechanical indexes after salt washing.

The tested soil samples were taken from a subgrade engineering filler in the suburbs of Zhangye City along the Belt and Road in China (sampling longitude: 100.45°, latitude: 38.93°). The soil was washed with salt before the test. The salt-washing process is as follows: pour the crushed soil into the bucket, and then add the pure water and fully stir the soil and water. After standing for 24 h, the upper water should be drawn out. In order to ensure the accuracy of the test, the above process must be repeated 3∼4 times. After the salt washing process, the soil must be spread and dried, and the indoor geotechnical test must be *carried out in strict accordance with the Highway Geotechnical Test rules (JTG3430-2020)* ^18^*.* The test results are shown in Fig. 1, the maximum dry density is 1.78 g/cm^3^, and the optimal moisture content is 13.7%.

### Design of the test scheme

According to the provisions of *the Code for Design of High-speed Railway (TB 10621-2014)* ^19^ and *the Specifications for Design of Highway subgrade (JTG D30-2015)* ^20^, the compacted moisture content of the roadbed fill adopts the optimal moisture content. The degree of compaction is 96%. Because undrained road bed consolidation deformation will occur under long-term traffic load, undrained triaxial road construction deformation is suitable to be selected as the test method. The confining pressure of the triaxial test is designed as 100 kPa. The loading mode is graded loading. The test scheme is shown in Table 1.

**Table 1:** Creep test scheme for triaxial CU.

Number	Degree of compaction/%	confining pressure/kPa	NaCl Concentration/(mol/kg)	Saturation/%	Loading stress/kPa	Loading time/h
N1	96	100	0.50	60.07	50/100/150/200	24/24/24/24
N2	96	100	1.00	58.57	50/100/150/200	24/24/24/24
N3	96	100	2.00	57.14	50/100/150/200	24/24/24/24

Soil samples in the test scheme are all unsaturated saline soil.

**Figure 2:**
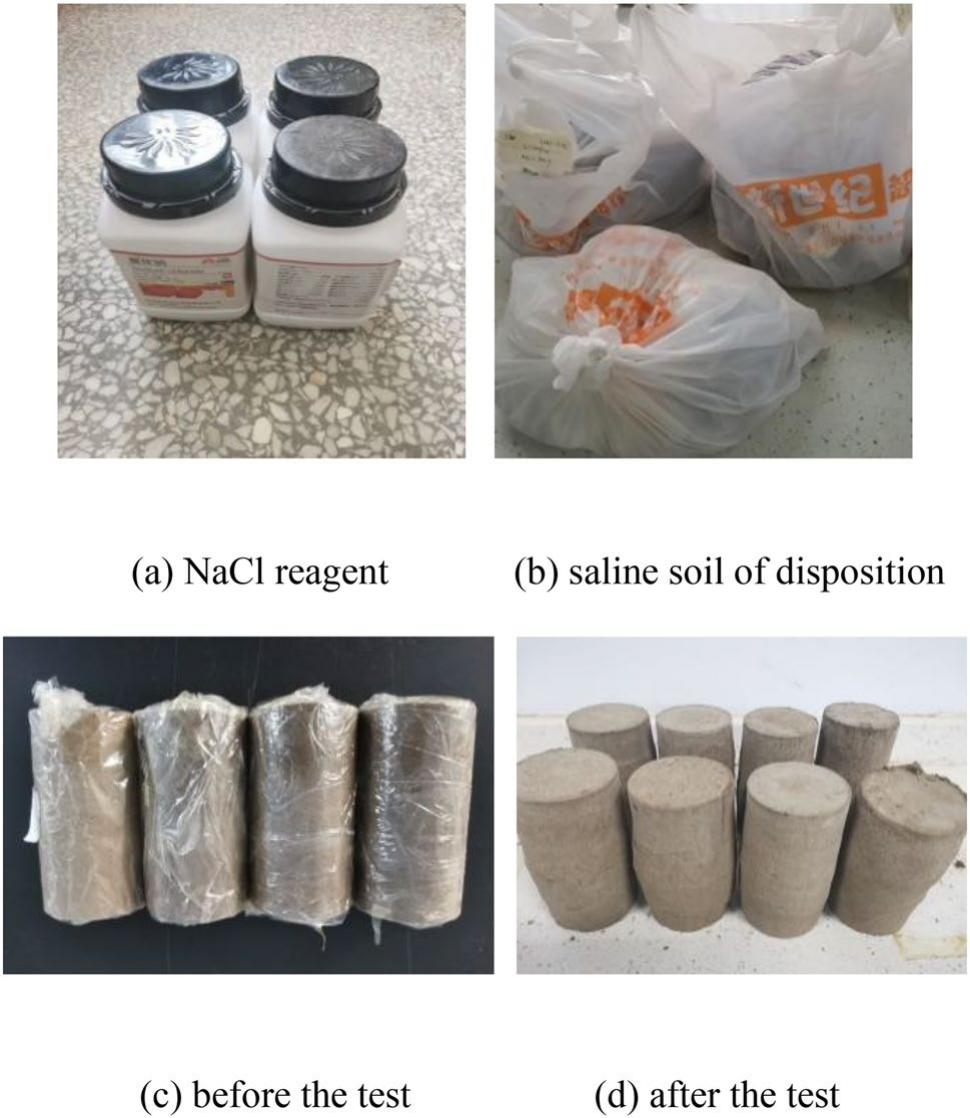
Test materials and sample preparation.

In strict accordance with the test plan in Table 1, the target NaCl solution was sprayed to make the soil absorb the moisture and a well-proportioned allocation (Sodium chloride reagent as shown in Fig. 2a). Sample preparation was carried out after bagging, sealing, and placing for 5 days ensure that the soil solution was well distributed, as shown in Fig. 2b. To ensure the uniformity of the compacted sample, the remolded saline soil was divided into five layers and compacted into a three-axis mold. The sample was a cylinder with a diameter of 61.8 mm and a height of 125 mm, as shown in Fig. 2c. In order to avoid delamination, the surface of the soil layer should be scraped. After the samples were prepared, they were wrapped with a cling film and sealed in plastic bags for 24 h before the triaxial CU creep test. After the test, the deformation of the saline soil sample was drum-shaped damage as shown in Fig. 2d.

### Test Procedure

**Figure 3:**
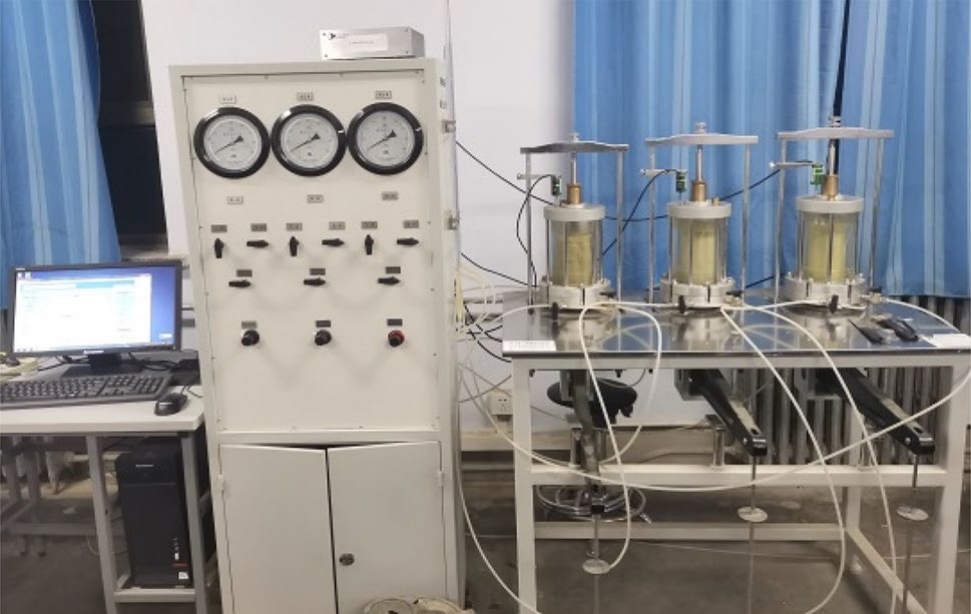
SR-6 triaxial creep system.

*Triaxial CU creep test* the test instrument adopts SR-6 triple triaxial creep tester, which includes triaxial pressure chamber, lever loading table, confining pressure control cabinet and data acquisition system. As shown in Fig. 3. The test steps are carried out in three steps:

① *Installation of samples* Wrap the sample in latex film → Placed on the base of the pressure chamber (filter paper and permeable stone should be added to the upper and lower contact surfaces) → Tighten the latex film under the soil sample → Latex film exhaust → Install the pressure cap at the top of the sample → Fasten the latex film on top of the soil sample → Lower the top cover of the pressure chamber and tighten the screws → Install vertical deformation sensor → Add water to the pressure chamber, Disassemble samples in reverse order.

② *Sample consolidation* open the confining pressure valve to increase the confining pressure to the consolidation confining pressure. In this test, the consolidation confining pressure is 100 kPa, open the drainage valve, carry out drainage consolidation, and the consolidation time is set to 60 min.

③ *Loading creep* keep the confining pressure when the consolidation is completed, close the drainage valve, and start the consolidation undrained triaxial creep test by applying design stress in stages by calibrated weights.

## Results of the triaxial CU creep test

### Analysis of strain–time curve

**Figure 4:**
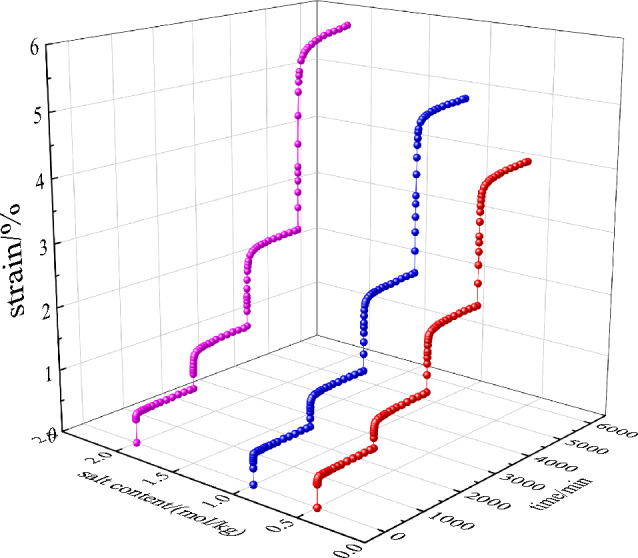
Triaxial CU creep curves under different salt content (K = 96%).

According to the experimental scheme, the unsaturated saline soil samples with the 96% compactness, whose NaCl concentrations are 0.50, 1.00, 2.00 mol/kg and the compaction is 96%, are subjected to a triaxial CU creep test under, and the full process curves of triaxial CU creep are obtained as shown in Fig. 4.

**Figure 5:**
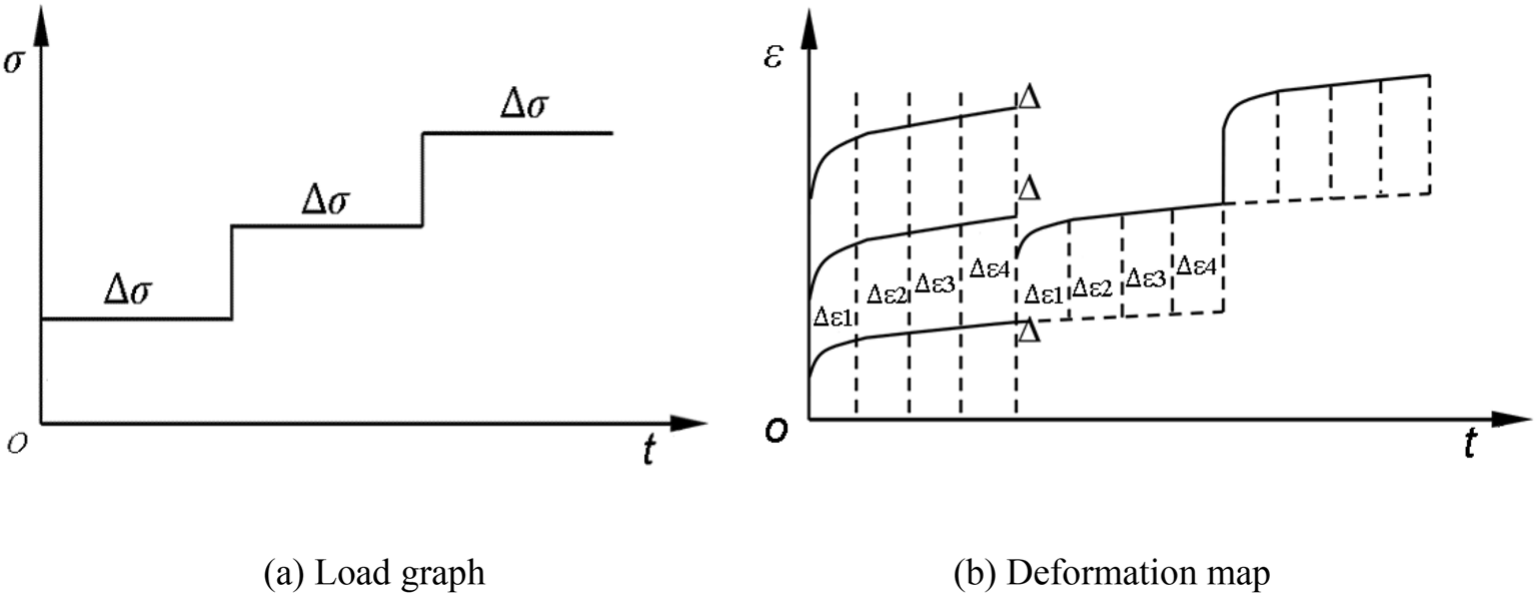
Principle of Chen's loading method.

**Figure 6:**
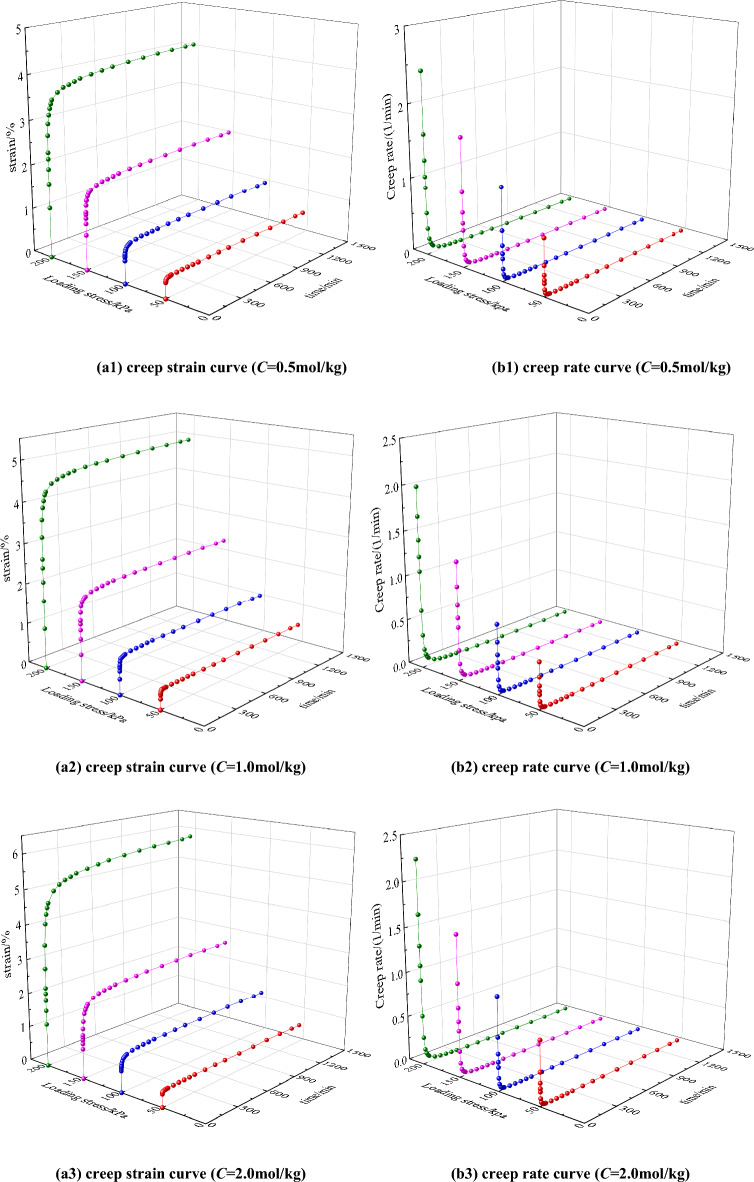
Creep strain and creep rate curves.

Chen's loading method was adopted to process the creep curve of the whole process under graded loading^21^. Its schematic diagram is shown in Fig. 5. Suppose that *t*_0_ is the start time of the stress loading and *t*_1_ is the time when the specimen enters the steady-state creep stage under this stress level. When the test progresses to *t*_1_, the sample deformation will continue along the dotted line if the lower stress is not applied. Therefore, the effect of applying the next level of stress on the specimen is the additional deformation between the dotted line and the solid line. The additional deformation is divided into (∆*ε*_1_ − ∆*ε*_n_) according to time (*t*_0_ − *t*_1_), and the axial strain-time curve under the action of the second order stress can be obtained by moving it and superimposing it to the time of the first order stress (*t*_0_ − *t*_1_). As shown in Fig. 5b. By that analogy, the loading creep curves under all levels of stress can be obtained, as shown in Fig. 6a1∼a3.

Figure 6 shows the creep strain and creep rate curves at different salt contents. It can be seen from Figs. 4 and 6 that unsaturated saline soil has obvious creep characteristics, and the creep curve develops in three stages: instantaneous creep stage, decelerated creep stage, and steady creep stage. At the initial stage of stress application, the axial strain increases sharply, and the larger the axial stress, the larger the axial strain, and this process is the transient creep phase; then enters the deceleration creep phase, the axial strain increases continuously with time, but the creep rate decreases continuously; finally enters the steady-state creep phase, the creep rate tends to 0, and the axial strain tends to a constant value. The triaxial creep deformation shows obvious creep decay characteristics and strong viscous properties.

As can be seen in Fig. 6, the creep behavior of saline soils is closely related to the loading stress and the salt content, and the creep amount increases gradually with increasing the loading stress under the same salt content. Under the same loading stress conditions, with increasing salt content, the creep amount increases and the time required to reach the steady-state creep stage is longer. The analysis mechanism is as follows: the skeletal structure of saline soils is stacked and like a honeycomb, and with increasing salt content, the more chaotic and loose the arrangement of crystalline salts in the pores, the soil particles and salt crystals change from face-to-face contact and line-to-surface contact to point-to-surface contact, and this change of microstructure leads to the decrease in mechanical properties and strength ^22^. At the same time, when the saline soil is subjected to load, the original balance of saline solution inside the soil is broken that resulting in a new ionization balance and ion interaction between the pore saline solution and the soil particles, and resulting in a chemical-mechanical coupling effect. Therefore, the higher the salt concentration, the stronger the coupling effect and the corresponding solution suction is also stronger, which will cause the effective stress to decrease ^14^.

### Analysis of the stress–strain isochronous curve

**Figure 7:**
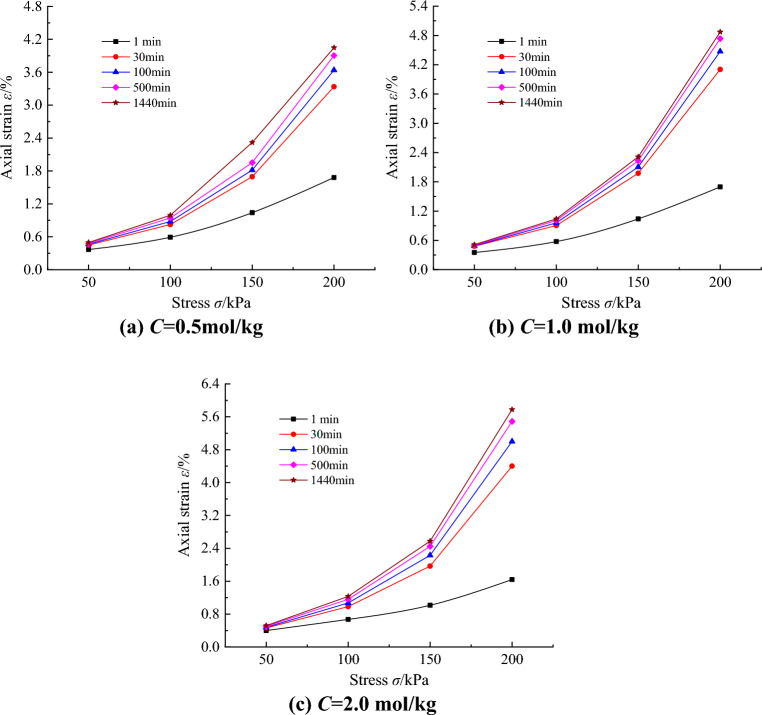
Stress-strain isochronous curves under different salt contents.

**Figure 8:**
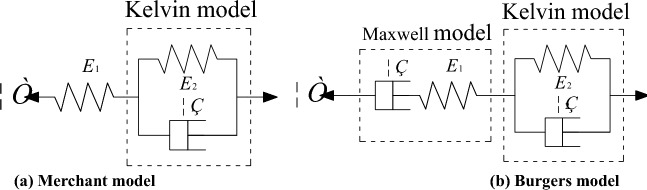
Component model diagram.

In order to further study the creep behavior of saline soil and understand the stress-strain variation law of saline soil in the creep process, the stress-strain data at different times are selected to obtain the stress-strain isochronous curve of unsaturated saline soil, as shown in Fig. 7. The results of the triaxial CU shear test for saline soils are shown in Fig. 8.

As can be seen in Fig. 7, the stress-strain isochronous curves at different salt contents develop similarly; with the accumulation of time, the stress-strain isochronous curves of saline soils gradually shift toward the strain axis, showing a nonlinear growth of curve clusters from dense to sparse, and there are obvious inflection points on the curve clusters, and the loading stress at the inflection points is the long-term strength of saline soils, with values ranging from 100 to 150 kPa. The creep deformation of saline soil accelerates with increase of loading stress.

## Creep model analysis and parameter identification

Currently, there are empirical models and component models to describe the creep properties of the soil. For the creep deformation of the CU of unsaturated saline soil in Hexi under triaxial stress, the component models Merchant and Burgers are used for the identification and analysis of parameters in this paper, and the schematic diagram of the model is shown in Fig. 8.

### Merchant model

The Merchant model consists of a Hooke model in series with the Kelvin model ^23^, and the equations of the Merchant model for the triaxial stress state are

1 $$\varepsilon \left( t \right){ = }\varepsilon_{1} \left( t \right) + \varepsilon_{2} \left( t \right){ = }\frac{{\sigma_{1} - \sigma_{3} }}{{E_{1} }} + \frac{{\sigma_{1} - \sigma_{3} }}{{E_{{2}} }}\left( {{\text{1 - e}}^{{{ - }\frac{{E_{{2}} }}{{\eta_{1} }}t}} } \right)$$

where $\varepsilon$ is the total strain; $\sigma$ is the loading stress, kPa; $t$ is the loading time, min; $E_{1}$ is the elastic modulus of Hooke's law, kPa; $E_{2}$ is the elastic modulus of the Kelvin model, kPa; $\eta_{1}$ is the viscosity coefficient of the Kelvin model, kPa min.

### Burgers model

The Burgers model is made up of the Maxwell and Kelvin model in series ^24^. Burgers model can better describe the whole process of instantaneous elastic deformation, deceleration creep, and stable creep of rock and soil. The differential equation is the following:

2 $$E_{1} \varepsilon + \eta_{1} \varepsilon { = }\frac{{\eta_{1} }}{{E_{0} }}\sigma + \left( {1 + \frac{{E_{1} }}{{E_{0} }} + \frac{{\eta_{1} }}{{\eta_{0} }}} \right)\sigma + \frac{{E_{1} }}{{\eta_{0} }}\sigma$$

The Burgers model equation for the triaxial creep stress state can be expressed as ^25^

3 $$\varepsilon \left( t \right){ = }\frac{{\sigma_{1} - \sigma_{3} }}{{3E_{1} }} + \frac{{\sigma_{1} - \sigma_{3} }}{{3\eta_{1} }}t + \frac{{\sigma_{1} - \sigma_{3} }}{{3E_{{2}} }}\left( {{\text{1 - e}}^{{ - \frac{{E_{{2}} }}{{\eta_{{2}} }}t}} } \right)$$

where $\varepsilon$ is the total strain; $\sigma$ is the loading stress, kPa; $t$ is the loading time, min; $E_{1}$ is the elastic modulus of the Maxwell model, kPa; $\eta_{1}$ is the viscosity coefficient of the Maxwell model, kPa min; $E_{2}$ is the elastic modulus of the Kelvin model, kPa; $\eta_{2}$ is the viscosity coefficient of the Kelvin model, kPa min. min.

### Identification of parameters of the Merchant and Burgers model

Currently, there are two methods to obtain the parameters of the creep model: (1) forward thinking in which the solution is based on the test results and mechanical theory; and (2) inverse thinking in which the parameters are inverted using mathematical methods such as least squares in the test data ^26^. In this paper, the second reverse thinking method is used to realize the inversion and fitting analysis of model parameters by Levenberg-Marquardt optimization algorithm based on origin software, and the parameters of the Merchant and Burgers model and the correlation coefficients of the saline soil in Hexi are obtained, as shown in Tables 2 and 3.

**Table 2:** Parameters of the Merchant creep model.

Number	NaCl concentration/(mol/kg)	Loading stress/kPa	*E*_1_ (kPa)	*E*_2_ (kPa)	*η*_1_ (kPa min)	R^2^
N1	0.5	50	2.49E+05	11,286.682	6396.895	0.886
		100	7.94E+04	12,919.897	19,401.283	0.849
		150	6.79E+04	9118.541	18,044.368	0.900
		200	4.91E+04	6004.203	16,549.166	0.924
N2	1	50	2.19E+05	10,729.614	10,080.907	0.960
		100	1.48E+05	11,025.358	16,885.197	0.937
		150	1.01E+05	7496.252	15,589.261	0.943
		200	5.06E+04	4803.074	15,855.916	0.950
N3	2	50	5.31E+05	10,570.825	4698.416	0.905
		100	4.69E+04	11,210.762	23,644.414	0.823
		150	1.85E+04	9345.794	160,058.133	0.900
		200	1.60E+04	4820.439	73,281.220	0.946

**Table 3:** Parameter values of the Burgers creep model.

Number	NaCl concentration/(mol/kg)	Loading stress/kPa	*E*_1_ (kPa)	*η*_1_ (kPa min)	*E*_2_ (kPa)	*η*_2_ (kPa min)	R^2^
N1	0.5	50	2.37E+05	3.48E−09	3858.02	1571.13	0.937
		100	4.26E+04	4.83E−09	4450.38	4591.62	0.923
		150	3.27E+04	6.08E−09	3172.59	4794.17	0.954
		200	2.42E+04	8.80E−09	2087.25	4443.60	0.965
N2	1	50	1.00E+05	2.26E−09	3654.97	3049.85	0.977
		100	7.07E+04	3.64E−09	3813.88	4908.79	0.972
		150	4.95E+04	5.73E−09	2602.81	4520.10	0.976
		200	2.51E+04	8.93E−09	1659.61	4445.08	0.976
N3	2	50	5.41E+05	3.74E−09	3663.00	1253.73	0.964
		100	2.56E+04	6.76E−09	3831.42	4935.87	0.909
		150	1.06E+04	8.79E−09	3015.68	12,936.18	0.935
		200	7.43E+03	1.30E−08	1677.99	12,627.88	0.969

As can be seen in Table 2, the correlation coefficients obtained by fitting the creep properties of unsaturated saline soils in the Hexi corridor using the Merchant model vary widely, range is 0.832∼0.960. When the salt content is the same, the elastic modulus *E*_1_ in the Hooke model decreases gradually with the increase of loading stress, which is similar to the conclusion of muddy silty clay in Ref. ^27^ and it reflects the instantaneous stiffness characteristics of soil, which is the instantaneous elastic deformation at the moment of loading. The spring stiffness *E*_2_ in the Kelvin model first increases and then decreases with the increase of loading stress. When the loading stress is 100 kPa, the spring stiffness *E*_2_ reaches the peak value, at the same time, the loading stress is equal to the confining pressure, which is the deformation of the soil in the creep process. Under the same salt content, the correlation between the viscosity coefficient *η*_2_ and the loading stress in the Kelvin model is not obvious, which is similar to the conclusion of the clay in Ref. ^28^. With the increase of salt content, the average value of *E*_1_ under each loading stress gradually increases, and the average values of *E*_2_ and *η*_2_ first decrease and then increase, indicating that the Merchant model parameters are greatly affected by the salt content of saline soil.

As can be seen in Table 3, the correlation coefficients obtained by fitting the creep properties of unsaturated saline soils in the Hexi corridor using the Burgers model are greater than those of the Merchant model, indicating that the fit accuracy of the Burgers model is higher than that of the Merchant model. Under the same salt content, the elastic modulus *E*_1_ and *E*_2_ in Burgers model increased and then decreased with increasing loading stress, and *E*_1_ and *E*_2_ reached the peak when the loading stress was equal to the surrounding pressure (100 kPa); while the viscosity coefficients *η*_1_ and *η*_2_ are affected by the test conditions, the consolidation method and sample making method ^27^, and the correlation between *η*_1_ and *η*_2_ with loading stress is not obvious. With the increase of salt content, the average values of *E*_1_, *E*_2_ and *η*_1_ under all levels of loading stress decrease initially and increase afterwards, and the average value of *η*_2_ increases.

### Comparative analysis of the Merchant and Burgers models

**Figure 9:**
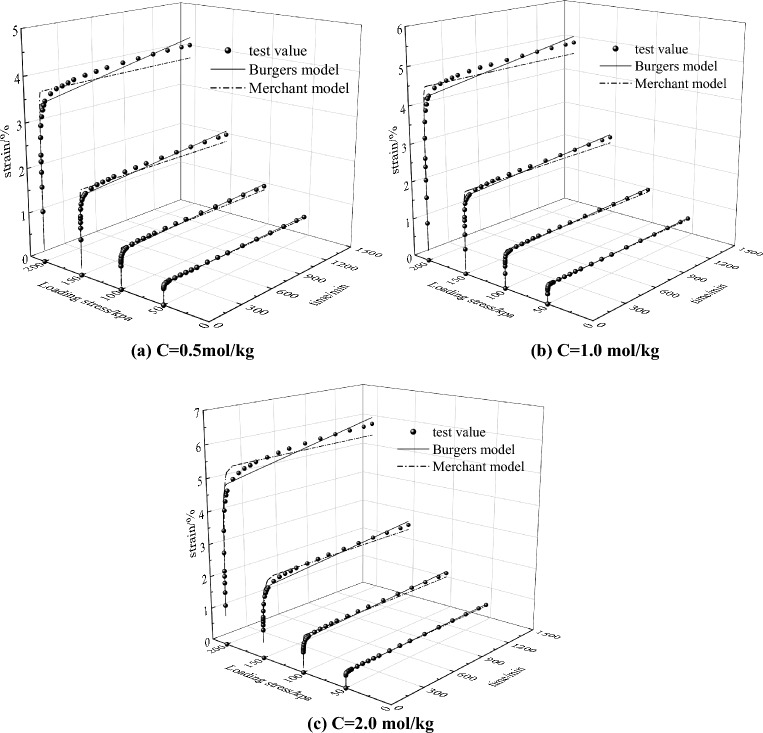
Comparison results of model calculation curves and test values under different salt contents.

In order to further verify the applicability of Merchant and Burgers model to saline soil in the Hexi Corridor. the values of the model parameters in Tables 2 and 3 were substituted in Eqs. (1) and (3) for the calculation, and the calculated results were compared and analyzed with the results of the triaxial CU creep tests on saline soils, and the results are shown in Fig. 9.

From Fig. 9, it can be seen that both the Merchant model and Burgers model can predict the creep behavior of saline soils in the Hexi corridor under different salt contents well. When the loading stress is 50 kPa and 100 kPa, the calculated values of the Merchant and Burgers models agree highly with the experimental measured values, and both models can describe the creep deformation law the of saline soil well; When the loading stress is 150 kPa and 200 kPa, the calculated results of the Merchant model are slightly higher than the experimental results at the beginning and with the increase of time the calculated results; When the loading stress is 150 kPa and 200 kPa, the calculation result of the Merchant model is slightly higher than the test result at the beginning, and slightly lower than the test result as the time increases. In order to discriminate the prediction accuracy of Merchant model and Burgers model more precisely, the relative error formula is used in this paper as follows.

4 $$S_{{{\text{err}}}} = \sqrt {\frac{{\mathop \sum \limits_{i = 1}^{n} (N_{p,i} - N_{m,i} )^{2} }}{{\mathop \sum \limits_{i = 1}^{n} N_{m,i}^{2} }}}$$

where $S_{{{\text{err}}}}$ is the relative error value; $N_{p,i}$ is the *i* predicted value, kPa; $N_{m,i}$ is the *i* measured value, kPa.

Table 4 shows the results of the accuracy of the Merchant and Burgers model prediction. From Table 4, it can be seen that the relative error calculation results of Burgers model under different salt content and different loading stress conditions are smaller than those of the Merchant model. The results show that the prediction results of Burgers model are closer to the experimental values, and the prediction accuracy is better, which is more suitable for describing the creep behavior of the unsaturated saline soil in Hexi Corridor. Therefore, in view of the actual engineering problems such as the engineering design, reinforcement and post-construction settlement prediction of unsaturated saline soil slope, subgrade and foundation in Hexi Corridor, the Burgers model can be used in the establishment of finite element model analysis, and the model parameters can be selected by referring to Table 3 for value calculation.

**Table 4:** Prediction accuracy of the Merchant and Burgers models.

NaCl concentration/(mol/kg)	Model type	Loading stress/kPa			
		50	100	150	200
0.5	Merchant	0.0735	0.0986	0.0873	0.0841
	Burgers	0.0547	0.0703	0.0593	0.0565
1.0	Merchant	0.0465	0.0664	0.0694	0.0726
	Burgers	0.0351	0.0443	0.0448	0.0506
2.0	Merchant	0.0655	0.1125	0.1020	0.0851
	Burgers	0.0401	0.0811	0.0828	0.0647

## Conclusion

1. Unsaturated saline soils have significant creep characteristics, and the creep process goes through the transient creep phase, deceleration creep phase, and steady-state creep phase; the creep behavior shows obvious creep decay characteristics and strong viscous characteristics.
2. Salt content and loading stress have a large influence on creep properties of unsaturated saline soils. The salt content and loading stress affect the skeletal structure, microcontact and effective stress of saline soils. As the salt content increases, the solution of the suction increases, the effective stress decreases, and the structure of the honeycomb becomes more obvious, leading to the decrease in the mechanical properties and strength of saline soils.
3. The isochronous stress-strain curve of unsaturated saline soils shows a nonlinear growth of the curve cluster, with a shift toward the strain axis as time increases, and there are visible inflection points on the curve cluster, and the stress at the inflection point is the long-term strength of the saline soil.
4. The salt content and loading stress have a greater influence on the parameters of the Merchant and Burgers models. The prediction accuracy of Burgers model is higher than that of the Merchant model under different salt content and loading stress conditions. Therefore, the Burgers model is more suitable for predicting the creep behavior of unsaturated saline soils in the Hexi corridor.

## Data Availability

The data produced and analyzed during the current study are available from the corresponding author on reasonable request.

## References

[1] Yl X (2019). The changing process and trend of ground temperature around tower foundations of Qinghai-Tibet Power Transmission line. Sci. Cold Arid Regions.

[2] Wan X, Liu E, Qiu E (2020). Study on phase changes of ice and salt in saline soils. Cold Reg. Sci. Technol..

[3] Venda Oliveira PJ (2019). Numerical prediction of the creep behaviour of an embankment built on soft soils subjected to preloading. Comput. Geotech..

[4] Zhu C, Li N (2020). Ranking of influence factors and control technologies for the post-construction settlement of loess high-filling embankments. Comput. Geotech..

[5] Yuan Y, Liu R, Qiu CL (2018). Establishment and application of creep constitutive model related to stress level of soft soil. J. Tianjin Univ. (Sci. Technol.).

[6] Wang SH, Luo YS (2010). Triaxial shear creep characteristics of loess. Chin. J. Geotech. Eng..

[7] Liu X, Zhang X, Fu X (2022). Experimental study on creep characteristics of saturated Q2 loess. Front. Earth Sci..

[8] Cai G, Chen S, Zhao D (2019). Experimental study on creep characteristics of saturated sand and its influencing factors. J. Eng. Geol..

[9] Wang PC, Luo YS, Hu LX (2015). Research on triaxial creep characteristics and models of remolded loess. Rock Soil Mech..

[10] Shan S, Xie WL, Zhu RS (2021). Study on creep characteristics of compacted loess in Yan'an new area under loading and unloading. Resour. Environ. Arid Area.

[11] Xue K, Wang S, Hu Y (2020). Creep behavior of red-clay under triaxial compression condition. Front. Earth Sci..

[12] Long Z, Cheng Y, Yang G (2020). Study on triaxial creep test and constitutive model of compacted red clay. Int. J. Civ. Eng..

[13] Wang Y, Cong L, Yin X (2021). Creep behaviour of saturated purple mudstone under triaxial compression. Eng. Geol..

[14] Zhou FX, Wang LY, Lai YM (2020). One dimensional creep test and model study of saturated saline soil. Chin. J. Geotech. Eng..

[15] Wang LY, Zhou FX, Qin H (2020). Fractional creep model and experimental study of saturated saline soil. Rock Soil Mech..

[16] Niu FJ, Lin ZJ, Lu JH (2011). Study of the influencing factors of roadbed settlement in embankment-bridge transition section along Qinghai-Tibet Railway. Rock Soil Mech..

[17] Yaling C, Shuangshuang Y (2023). Research progress on the improvement of saline soil engineering properties. Mater. Rep..

[18] JTG3430-2020 (2020). Test Methods of Soils for Highway Engineering.

[19] TB 10621-2014 (2014). Code for Design of Speed Railway.

[20] JTG D30-2015 (2015). Specifications for Design of Highway subgrade.

[21] Liu X (1994). An Introduction to Rock Rheology.

[22] Wang XG (2021). Research on Microstructure and Engineering Characteristics of Alar Saline Soil in Southern Xinjiang.

[23] Bялoв CC (1987). The Rheological Principle of Soil Mechanics.

[24] Mansouri H, Ajalloeian R (2018). Mechanical behavior of salt rock under uniaxial compression and creep tests. Int. J. Rock Mech. Min. Sci..

[25] Zhang ZL, Xu WY, Wang W (2011). (2011) Study of triaxial creep tests and its nonlinear visco-elastoplastic creep model of rock from compressive zone of dam foundation in Xiangjiaba hydropower station. Chin. J. Rock Mech. Eng..

[26] Huang YQ (2010). Study on Highway Subgrade Settlement and Pavement Dynamic Characteristics.

[27] Deng HY, Dai GL, Qiu GY (2021). (2021) Drained creep test and component creep model of soft silty clay in Hangzhou Bay. J. Southeast Univ..

[28] Kong LW, Zhang XW, Guo AG (2011). (2011) Creep behavior of Zhanjiang strong structured clay by drained triaxial test. Chin. J. Rock Mech. Eng..

